# *In vitro* characterization of fiber-rich tropical feedstuffs for pig diets

**DOI:** 10.5455/javar.2025.l995

**Published:** 2025-12-25

**Authors:** Quan Hai Nguyen, Phung Dinh Le, Ngoan Duc Le

**Affiliations:** Department of Animal Science, University of Agriculture and Forestry, Hue University, Hue City, Vietnam

**Keywords:** Ammonia, Fiber-rich feedstuffs, *In vitro*, Volatile fatty acid

## Abstract

**Objective:**

This study aimed to characterize the characteristics of fiber-rich feedstuffs using a three-step *in vitro* method.

**Materials and methods:**

A three-step *in vitro* digestibility simulation included step 1 and step 2, which were referred to as “enzymatic hydrolysis” and mimicked processes in the stomach, followed by an ileal digestibility assessment, whereas step 3 mimicked the hindgut fermentation using fecal inoculum. The fiber-rich feedstuffs used were banana stem (*Musa acuminata*) (Bas), brewery by-product (Brew), cassava (*Manihot esculenta*) leaf (CL) and root by-product, cabbage waste (*Brassica oleracea*) (Caw), sweet potato vines (*Ipomoea batatas*) (Swe), taro leaves and petioles (*Colocasia esculenta*) (Taro), tofu by-product (TF), and *Trichanthera gigantea* foliage (Tri).

**Results:**

Tri and TF were marginally (10%) digested in the small intestine; for TF, this was largely compensated for by the level of the hindgut, resulting in the highest volatile fatty acid (VFA) production from TF as compared with any other fiber-rich feedstuff tested. Nevertheless, 55% of the crude protein (CP) of TF was digested in the small intestine, resulting in a modest accumulation of hindgut ammonia as compared with CP from Tri, which particularly seemed to be fermented in the hindgut. For the complete diets, the ileal digestible CP of the test diets was 7.8% to 18.5% lower than that of the control diet, except for the TF diet. Only diets including Bas and CL enhanced hindgut VFA production compared with the control.

**Conclusion:**

Fiber-rich feedstuffs differ widely in intestinal digestibility and hindgut fermentation. The three-step *in vitro* simulation results for the nine tested fiber-rich feedstuffs could provide valuable insights into assessing feeding values for pig production. Additionally, an interaction was observed between fiber source and dietary nutrients in complete diets, which impaired ileal CP digestibility.

## Introduction

Increasing dietary fiber has been receiving attention as it is effective in improving pigs’ health and welfare [1]. Moreover, dietary fiber has been reported as a nutritional strategy that may reduce ammonia emissions [2]. In this respect, valorization of such agricultural and industrial by-products, as well as fibrous green plants derived from non-agricultural land, is of interest.

However, the use of fiber in monogastric diets is a topic of controversy. Indeed, both positive effects (e.g., reducing ammonia emission, stimulating gut health, and improving animal well-being) and negative effects (decreasing nutrient utilization, low net energy values) have been reported [3–5. Pu et al. [6] reported that short-term feeding of a high-fiber diet did not improve animal performance but enhanced fiber digestion and microbial diversity.

Nevertheless, fibrous resources differ considerably in their effects on digestibility and nutrient supply, which depend on fiber characteristics such as soluble and insoluble components [7]. However, even detailed chemical characterization of these dietary fiber sources does not allow an accurate prediction of their digestibility and interaction with other nutrients. Therefore, *in vitro* simulation of digestive processes is of interest to accurately characterize dietary fiber, both when incubated alone and when incorporated into complete diets, to assess potential interactions with other nutrients. Moreover, the *in vitro* setup has been confirmed as a suitable approach for studying dietary fiber in pig nutrition [8,9] and, in particular, for assessing odor and ammonia emissions prior to testing*in vivo* [10].

The current study was conducted to (1) screen the digestibility and fermentation capacity of nine individual fiber-rich ingredients, both individually and in combination within complete diets, through *in vitro* simulation of enzymatic hydrolysis in the stomach and small intestine, as well as hindgut fermentation and (2) test the potential interactions between fiber sources and other dietary nutrients in complete diets.

## Materials and Methods

### Ethical approval

This study did not involve live animals or human participants. All experimental work was conducted exclusively in the laboratory; therefore, ethical approval was not required.

### Ingredients

Nine feed ingredients with at least about 350 gm total dietary fiber (Table 1) with potentially high fermentability were studied: banana stem (*Musa acuminata*) (Bas), brewery by-product (Brew), cassava (*Manihot esculenta*) leaf (CL) and root by-product (CR), cabbage waste (*Brassica oleracea*) (Caw), sweet potato vines (*Ipomoea batatas*) (Swe), taro leaves and petioles (*Colocasia esculenta*) (Taro), tofu by-product (TF), and *Trichanthera gigantea* foliage (Tri). Bas, CL, Swe, Taro, and Tri are green plants or parts thereof that were collected from three farms in the villages near Hue City, Vietnam. They were harvested and sundried. TF was collected from three tofu processing households in Hue City, Vietnam. The TF was collected from the following process: soybeans were dehulled, ground, and soaked in water. Next, the solution was centrifuged to recover the soy milk. The solid residue from this step was the fresh TF, which was then sundried. Cabbage leaves represented the fresh outer leaves, which are generally discarded, and were obtained from three local vegetable farmers at the harvest of the cabbage. The leaves were sun-dried. Three batches of CR and Brew were collected from three different locations of local factories: TINH BOT SAN (Phong Dien district, Hue city, Vietnam) and HUDA BEER (Hue City, Vietnam), respectively. The CR refers to the solid fibrous residue that remains after the starch content has been extracted from cassava roots, while Brew is spent grain from the filtration process of beer production. Three batches of five popular ingredients of pigs’ diets as sources of starch and protein, that is, cassava powder, rice bran, maize, soybean meal, and fishmeal, were collected in a local feedstuffs store. Both the nine test ingredients and the five common feed ingredients were chemically characterized and assessed through an *in vitro* simulation system. Samples were dried at 60°C for 24 to 48 h, after which the three samples of each ingredient were pooled and stored until further analysis and *in vitro* experiments.

**Table 1.** Chemical composition of ingredients (gm/kg DM), except for DM (gm/kg fresh matter).

Ingredient	Group	DM	Ash	CP	EE	ADF	ADL	IF	SF	TDF	NSP	S&S
Swe	Green plant	104	176	214	36.7	461	194	406	67.0	473	279	32.5
Taro		69.0	198	226	61.5	264	103	245	141	386	297	44.3
Tri		103	261	174	39.6	394	142	394	8.00	402	270	55.4
Brew	Industrial by-product	224	35.1	338	98.0	223	63.5	452	6.90	459	312	78.4
CR		122	17.4	26.9	3.10	272	57.7	342	94.4	436	396	422
TF		83.1	31.9	162	60.9	274	17.7	672	15.5	688	627	26.9
Bas	Agricultural by-product	73.0	98.6	57.7	12.5	360	76.1	421	47.2	468	465	229
Caw		62.9	120	214	25.2	238	17.2	290	48.6	339	362	147
CL		275	62.9	315	92.2	271	89.7	411	17.8	429	352	19.3
Cassava powder	Common feed ingredients	882	16.9	34.7	8.5	46.8	8.30	71.6	21.9	93.5	80.7	756
Finely ground corn		965	13.9	89.7	57.5	38.8	5.10	133	4.10	137	245	517
Rice bran		931	64.3	138	150	91.9	1.10	158	41.2	199	189	388
Fishmeal		888	266	575	39.5	52.4	1.20	21.3	59.5	80.8	0.10	3.30
Soybean meal		944	77.4	480	29.5	81.1	10.6	198	76.9	275	198	120

Cassava powder = finely ground cassava root (https://www.feedipedia.org/); IF = insoluble fiber; S&S = total starch and sugar; SF = soluble fiber; TDF = total dietary fiber.

### In vitro procedure

A three-step *in vitro* digestibility simulation was modified from the technique described by Vervaeke et al. [11]. Steps 1 and 2 are referred to as “enzymatic hydrolysis” and mimic processes in the stomach, followed by an ileal digestibility assessment, whereas step 3 mimics the hindgut fermentation using fecal inoculum. The different steps are described in detail as follows.

### Enzymatic hydrolysis

For step 1, 2 gm of finely ground sample (<1 mm) was weighed in an Erlenmeyer flask. A 30 ml HCl 0.075 N was added to each flask, and then the pH was adjusted to pH 2 with 1 M HCl and 1 M NaOH solution. Then, 10 ml of a 0.8% pepsin (Porcine, 2000 FIP-U/gm, Merck No. 7190) solution dissolved in 0.075 M HCl was added. Flasks were closed with parafilm and placed in a shaking incubator at 39°C for 2 h. At the start of step 2, pH in each flask was adjusted to 7.5 using 1M NaOH. Then, 40 ml of pre-warmed and freshly prepared 1% pancreatin solution (10 gm pancreatin in 1 L of phosphate buffer) at pH 7.5 was added to each flask. Phosphate buffer was a mixture of 160 ml/l of 0.2 M NaH₂PO₄.H₂O and 840 ml/l of 0.2 M Na₂HPO₄.12H₂O. Flasks were closed with parafilm and placed in the shaking incubator at 39°C for 4 h. After enzymatic hydrolysis, the incubation content was quantitatively recovered in a glass centrifugation tube (the empty weight of the centrifugation tube was registered) to centrifuge at 2,000 gm for 10 min (Hermle, Germany). Supernatant was removed by pouring the glass tube through a nylon cloth (pore size of 37 µm) (Solana, UK). Afterward, 20 ml of distilled water was added to the centrifugation tube to wash the pellet, which was then centrifuged a second time. This washing step was repeated twice. The residue in the glass centrifugation tubes and nylon cloth was put in the oven at 60°C for 48 h and weighed (after cooling in a desiccator). Steps 1 and 2 of the *in vitro* procedure were repeated four times; all hydrolyzed residues were pooled for further analysis (N and gross energy) and used as a substrate for the fermentation step (step 3).

### In vitro fermentation

#### Preparation for inoculum

Feces from five donor pigs (Institute for Agricultural and Fisheries Research ILVO, Belgium) were collected directly from the rectum and then immediately placed in collection tubes (Falcon tubes), which were completely filled and closed airtight. Gestating sows were chosen as donor animals, as their diets contain somewhat more fiber than the diets of growing animals. The collection tubes were transported in thermos flasks filled with water at 39°C. Feces from the five donor animals were weighed and mixed in equal portions in a beaker under a permanent flux of CO₂. Phosphate-bicarbonate buffer (3.58 gm/l Na₂HPO₄.12H₂O, 1.55 gm/l KH₂PO₄.H₂O, 0.124 gm/l MgCl₂.6H₂O, 8.74 gm/l NaHCO₃, and 1 gm/l NH₄HCO₃) was added, and the mixture was homogenized with a hand blender for 1 min. The mixture was then filtered through cheesecloth, and then, the inoculum was adjusted with the phosphate-bicarbonate buffer to reach a dilution of 0.05 gm feces ml^−1^ buffer [12].

Two hundred milligrams of the substrate (residue of step 2) were added to each incubation flask (volume of 120 ml), together with 1 ml of distilled water. The incubation flasks were sealed with a rubber stopper and aluminum cap and attached to a vacuum gas filling system. Air was removed using the vacuum pump, after which the flask was filled with CO₂.

Afterward, 19 ml of the phosphate-bicarbonate buffer mixed with fecal inoculum was added to each flask. Flasks were placed in the incubator at 39°C and shaken regularly for 48 h.

Each pooled sample was incubated in triplicate, resulting in 45 bottles for the feed ingredients (14 ingredients and a blank without any substrate) and 33 bottles in the tests with the complete diets (10 diets and one blank).

### Calculations and diet formulation

#### ME calculation

The calculated Metabolisable Energy of ingredients is the sum of two parts: First, the amount of energy released during enzymatic hydrolysis was calculated as the difference between Gross Energy of dietary ingredients and the combustion energy of pooled residues. Second, volatile fatty acid (VFA) produced from the residues of the enzymatic hydrolysis was assumed to contribute to ME supply, which was estimated as described by Fievez et al. [13]. In which, combustion energies of 874, 1,535, and 2,192 (kJ mol^−1^) were assumed for acetate, propionate, and butyrate, respectively.

#### Ileal digestible crude protein and dry matter

These are the amounts of protein and dry matter (DM) lost through enzymatic hydrolysis, which were calculated as the difference between crude protein (CP) or DM of dietary ingredients and CP or DM of residues after steps 1 and 2 of the incubation.

#### Diet formulation

The *in vitro* experiment screened nine fiber-rich feedstuffs varying in chemical composition, particularly in terms of the total proportion of soluble and insoluble fiber and CP (Table 1). As the fiber fraction may also interact with the digestibility of other nutrients originating from other feed ingredients, possible interactions between fiber sources and other ingredients in complete diets were assessed by composing complete diets, which were then subjected to *in vitro* simulation. Complete diets were formulated based on common ingredients used in practical diets for growing pigs and including none (control diet) or one of the nine fiber sources (test diets) (Table 2). Furthermore, to potentially be of practical relevance, the test diets were formulated to achieve similar nutritive values in terms of ME content and digestible CP supply in the small intestine. For this purpose, diets were formulated using the Solver application in Excel to target non-starch polysaccharides (NSPs) levels of 150 gm/kg for the control diet and 200 gm/kg for the other nine test diets. Moreover, all diets were formulated to meet the following additional constraints: CP content between 160 and 170 gm/kg, IDCP between 120 and 125 gm/kg, ME content between 12.1 and 12.3 MJ/kg, and a contribution of NSP from the fibrous feed resources to the total dietary NSP of 15%. Given the large variation in chemical composition and nutritive values of the test sources, diets could only be formulated within these relatively narrow ranges, that is, it was practically impossible to increase the NSP content above 200 gm/kg DM while keeping the other characteristics, as mentioned earlier, equal. For the formulation of the complete diets, both the chemical characterization (Table 1) and the *in vitro* data of the individual feedstuffs were used.

**Table 2.** The proportions of ingredients in complete diets (CD) and their chemical composition.

Items	Control CD	Swe CD	Taro CD	Tri CD	Brew CD	CR CD	TF CD	Bas CD	Caw CD	CL CD
Test ingredient	-	111	109	114	95.1	80.5	50.6	66.2	84.5	84.9
Cassava powder	315	177	144	162	199	96.9	147	129	125	219
Finely ground corn	217	394	361	496	316	430	410	522	349	331
Rice bran	300	157	236	49.8	257	228	242	109	300	206
Fishmeal	93.4	21.7	8.50	34.8	0	79.4	73.2	96.5	35.5	0
Soybean meal	74.3	140	142	143	133	86	77.1	77.6	106	160
Chemical composition										
Dry matter	916	922	928	919	922	921	920	919	918	927
Crude protein	164	168	161	164	164	165	163	159	162	171
Metabolizable energy	12.2	12.1	12.3	12.1	12.3	12.3	12.2	12.3	12.3	12.3
NSP	150	200	200	200	200	200	200	200	200	200
IDCPa	125	125	123	122	122	126	123	120	124	123
ADF	61.7	101	83.1	89.8	77.2	75.2	69.0	71.5	77.5	78.0
ADL	4.95	26.6	16.0	21.7	11.0	8.90	5.38	9.83	5.77	13.0
Starch	476	419	409	422	437	428	428	434	416	437
Insoluble fiber	116	163	151	159	166	146	154	141	149	159
Soluble fiber	31.4	31.4	41.1	21.6	27.1	32.2	26.0	24.3	30.9	28.4
Total dietary fiber	147	194	192	181	193	179	180	165	180	187

Cassava powder = finely ground cassava root (Feedipedia.org).

aCalculated the IDCP values of the individual feeds as through *in vitro* simulation.

### Chemical analysis

The proximate compositions [DM, CP, ether extract (EE), and total ash] were analyzed using the Asscociation of Official Analytical Chemists procedure [14]. The gross energy of ingredients, diets, and enzymatic hydrolysis residues was determined by bomb calorimetry (Parr 1261 ISOPERIBOL, Moline, IL). Acid detergent fiber (ADF) was analyzed as a sequential analysis of the residual lignin and expressed inclusive of residual ash [15], and acid detergent lignin (pm) (ADL) was determined according to Van Soest et al. [15] by oxidation with permanganate. The insoluble fiber, soluble fiber, and total dietary fiber of feed ingredients were analyzed according to Prosky et al. [16]. The total sugar and starch content of feed ingredients was measured using phenol-sulfuric acid according to a protocol of Chow and Landhausser [17]. VFAs in the feces inoculum, as well as in the incubation fluid after step 3, were analyzed using a chromatographic method as described by Wambacq et al. [18], with Gas Chromatography specifications as described by Gadeyne et al. [19]. In the same samples, ammonia concentration was analyzed using the technique described by Nguyen et al. [20]. Finally, the NSP in feed ingredients was calculated as DM – crude ash – CP – EE – starch – (sugar × 0.965) – ADL [21].

### Statistical analysis

Ileal digestible DM (IDDM), ileal digestible energy (IDE), and ileal digestible CP (IDCP) after simulation of the stomach and small intestine digestion, as well as ammonia and VFA after fermentation, were analyzed using the one-way ANOVA procedure in SPSS (version 24.0). Each fiber-rich test ingredient or diet was the experimental unit.

*Y*_*ij*_=*μ + α*_*i*_ *+ e*_*ij*_

Where *Yij*=dependent variable;* μ* = the overall mean;*α*_*i*_=the fixed effect of treatment (*i* = ingredients or diets);* e*_*ij*_= the random error.

The Bonferroni post hoc test was used to compare nine fiber-rich test ingredients, and Dunnett’s post hoc test was used to compare the tested diets with the control diet when the *p*-value of the ANOVA test was < 0.05.

## RESULTS

### Ingredients

Tables 3 and 4 show the ileal digestibility of DM and CP, the amount of IDCP per weight unit of substrate, and the fermentation results obtained through *in vitro* incubation of the common feed ingredients and nine fiber-rich test ingredients, respectively. Brew contained almost twice the amount of IDCP (gm/kg DM) compared to CL (*p* < 0.05), although the total CP content of both ingredients was quite similar (Table 1), which was the highest (>300 gm/kg DM, Table 1) among all ingredients. The higher CP content was also reflected in the highest hindgut ammonia accumulation at around 300 mg/l compared to the other test ingredients (*p* < 0.05). On the other hand, Bas and CR contained the least amount of CP (<60 gm/kg DM, Table 1), consequently delivering the least IDCP and resulting in the lowest ammonia accumulation values compared to other ingredients (*p* < 0.05). Caw, Swe, and Taro had similar CP contents (around 210–220 gm/kg DM, Table 1), but Swe supplied considerably less IDCP due to its limited digestibility in the small intestine.

**Table 3.** Ileal digestibility of dry matter and crude protein, amount of IDCP, and fermentation results obtained through *in vitro* incubation of common feed ingredients.

Ingredient	IDDM (gm/gm)	IDCP (gm/gm CP)	IDCP (gm/kg DM)	Ammonia (mg/l)	Ammonia (mg/gm DM of feedstuff)	Ammonia (mg/gm CP of feedstuff)	Total VFA (µmol/incubation)	VFA (mmol/gm DM feedstuff)
Cassava powder	0.42	0.39	13.7	147	8.50	244	2051	5.96
Finely ground corn	0.38	0.65	58.1	188	11.6	129	1602	4.97
Rice bran	0.53	0.70	96.9	256	12.2	88.4	894	2.10
Fishmeal	0.52	0.82	531	696	33.2	51.3	335	0.80
Soybean meal	0.73	0.91	461	470	12.8	25.2	1200	1.64
SEM	0.02	0.01	1.01	11.0	0.88	10.1	130	0.51

Cassava powder = finely ground cassava root (https://www.feedipedia.org/).

**Table 4.** Ileal digestibility of dry matter and crude protein, amount of IDCP, and fermentation results obtained through *in vitro* incubation of fiber-rich ingredients.

Ingredient	IDDM1 (gm/gm DM)	IDCP2 (gm/gm CP)	IDCP (gm/kg DM)	Ammonia (mg/l)	Ammonia (mg/gm DM of feedstuff)	Ammonia (mg/gm CP of feedstuff)	Total VFA3 (µmol/incubation)	VFA (mmol/gm DM feedstuff)
Swe	0.21d	0.48d	102d	280ab	22.0bc	103cd	671bc	2.64cd
Taro	0.50a	0.62b	140c	292ab	14.6de	64.5cd	971b	2.42d
Tri	0.12e	0.17f	29.5f	315a	27.7a	160c	551c	2.42d
Brew	0.31bc	0.59bc	199a	326a	22.4bc	66.2cd	765bc	2.63cd
CR	0.38b	0.01h	0.27g	214c	13.4de	500a	1406a	4.37b
TF	0.10e	0.55c	89.8e	211c	19.0cd	117cd	1721a	7.74a
Bas	0.24cd	0.06g	3.18g	221c	16.8de	292b	913b	3.48bcd
Caw	0.50a	0.73a	156b	256bc	12.8e	60.0d	1498a	3.77bc
CL	0.25cd	0.34e	108d	334a	25.4ab	80.7cd	640bc	2.44d
SEM	0.02	0.01	1.83	11.0	0.97	19.5	66.0	0.23
*p*	<0.01	<0.01	<0.01	<0.01	<0.01	<0.01	<0.01	0.00

a–hMeans in the same column with different superscripts are significantly different (*p* < 0.05).

Nevertheless, the hindgut degradation of this ingredient is only modest, as indicated by VFA production, whereas relatively higher amounts of ammonia are accumulated. TF and Tri ingredients contained about 160 to 170 gm CP/kg DM, but IDCP (gm/kg DM) supplied by TF was triple the amount from Tri. Accordingly, intestinal residues were higher for Tri, resulting in higher levels of ammonia accumulation, whereas VFA production was the lowest among all test ingredients. TF produced the highest amount of hindgut VFA.

### Complete diets

Ileal digestibility of DM, energy, and CP; the amount of IDCP; and fermentation results obtained through *in vitro* incubation of complete diets are presented in Table 5.

**Table 5.** Ileal digestibility of dry matter, energy, and crude protein, amount of IDCP, and fermentation results obtained through *in vitro* incubation of complete diets (CD).

Diet	IDDM (gm/gm)	IDE (J/J)	IDCP (gm/kg DM)	Ammonia (mg/l)	Ammonia (mg/gm CD)	Ammonia (mg/gm CP of CD)	VFA (µmol/incubation)	VFA (mmol/gm CD)
Control CD	0.35	0.32	115	92.8	6.21	37.9	1,283	4.29
Bas CD	0.31	0.29	106*	99.8	6.77	42.6	1,577*	5.36*
Brew CD	0.33	0.31	100*	114*	7.69*	46.9*	1,421	4.78
CL CD	0.35	0.32	106*	101	6.60	38.6	1,500*	4.91
CR CD	0.32	0.31	106*	109	7.35	44.6	1,365	4.62
Caw CD	0.38	0.33	105*	109	6.67	41.2	1,428	4.38
Swe CD	0.33	0.31	105*	107	7.22	43.0	1,380	4.66
Taro C	0.37	0.34	104*	104	6.58	40.9	1,332	4.20
TF CD	0.31	0.29	111	90.0	6.15	37.8	1,401	4.80
Tri CD	0.29*	0.27	93.7*	113*	7.97*	48.6*	1,256	4.45
SEM	0.01	0.01	1.03	4.27	0.33	1.99	45.8	0.18

*Significantly different from the control treatment (*p* < 0.05).

The ileal digestibility of test diets did not differ from that of the control diet, except for the Tri diet, which showed lower ileal digestibility than the control diet (*p* < 0.05). All test diets showed a reduced amount of IDCP compared with the control diet, except for the TF diet, in which the amount of IDCP was maintained at the level of the control diet. The ammonia accumulation of Brew and Tri diets was higher than the control diet when expressed relative to the amount of original product or original CP of the product, as well as in mg/l. The other test diets did not differ from the control diet. The Bas diet produced more VFA than the control diet, both when expressed as mmol/gm of original diet and µmol/incubation. The CL diet produced higher amounts of total VFA (µmol/incubation) than the control diet.

## Discussion

The three-step *in vitro* setup was conducted to assess the enzymatic hydrolysis and fermentation capacity. The former was quantified through the parameters IDDM and IDCP, which are markers of ileal digestibility, and VFA production was used as an indicator for hindgut fermentative capacity. Ammonia accumulation in the hindgut simulation was monitored as a measure of balanced hindgut fermentation. Ammonia that accumulates in the in vitro system reflects excessive amounts of protein that are degraded compared to their incorporation into microbial biomass. Accordingly, lower levels of accumulating NH_3_ might originate from either a lower supply of protein to the hindgutdue to improved digestion in the small intestine and/or an enhanced sequestration of NH_3_ in microbial biomass. Both mechanisms potentially reduce ammonia emissions to the environment, while the former is additionally of interest for enhanced performance.

The first experiment was designed to screen various ingredients for their enzymatic hydrolysis and fermentation capacities. Ingredients differed widely in their chemical composition, particularly in CP content and fiber fractions. As the enzymatic hydrolysis, that is, the first step in the *in vitro* process, is based on pepsin and pancreatin enzymes, the fiber content remains undigested. This explained the low IDDM of TF, Tri, Swe, Bas, and CL given their high content of insoluble fiber and lignin (Table 1). Additionally, the IDCP varied between the fiber-rich feedstuffs, which may be linked to variable amounts of nitrogen bound to NDF, which becomes unavailable for enzymatic hydrolysis [22]. This has been reported to represent up to 31% for green plants and shrubs and 21% for grasses [23]. When the energy in the hindgut is depleted due to a lack of carbohydrate sources for fermentation, proteolytic fermentation may increase, resulting in a higher amount of ammonia produced. In the present results, Tri, Brew, and CL had the highest amount of ammonia accumulation when expressed in mg/gm DM and also had the highest amount of undigested CP (around 140–200 gm/kg DM). The result is in line with the findings of Jha et al. [24], who reported that the inclusion of fibrous feeds resulted in high amounts of indigestible nitrogen, contributing to N excretion. These authors also concluded that the mitigating effect of fermentable dietary fiber on ammonia mainly depends on the ratio of fermentable dietary fiber and IDCP. On the other hand, TF contains the highest amount of insoluble fiber (Table 1), which is the main carbohydrate source for fermentation in the hindgut. This, in combination with modest amounts of ileally undigested CP (around 70 gm/kg DM), might have stimulated a saccharolytic fermentation and limited a proteolytic fermentation, resulting in a low ammonia accumulation and the highest amount of VFA produced.

In the second experiment, the fiber-rich feedstuffs were included in complete diets, which were then evaluated through the same three-step *in vitro* setup. To potentially be of practical relevance, all diets were formulated to have the same amount of IDCP (gm/kg) based on the results of the enzymatic hydrolysis of the individual ingredients. However, the additivity of IDCP has been questioned when including fiber-rich feedstuffs into diets. Indeed, dietary fiber has been suggested to potentially reduce CP digestibility [25]. In the present experiment, the complete diets were not very different in soluble fiber, but the diets including the fiber sources contained higher amounts of insoluble fiber (from 25–50 gm/kg DM) compared to the control diet. That might be the main reason for reducing IDCP of the test diets compared to the control diet (except for the TF diet). This finding aligns with the results of Zhang et al. [25], who reported a reduction in IDCP of 0.5%–0.9% per unit per 100 gm increase in TDF.

As discussed earlier, the high ammonia accumulation was partially attributed to an increase in undigested N resulting from the lower IDCP (gm/kg). As such, protein supply to the hindgut was highest for the Brew and Tri diets. Both diets were also associated with higher ammonia accumulation, indicating the amount of fermentable carbohydrates was too limited to ensure an extensive bacterial nitrogen uptake, which was found to be associated with high VFA production [8,24]. The latter depended on the type of fibrous feed ingredients included in the diets. Part of the VFA production might originate from fermentable starch. Although the starch content was quite similar between test diets (Table 2), the ileal digestion of starch depends on its physicochemical structure [8,26], resulting in differences in the amount of fermentable starch supplied for hindgut fermentation. Hence, the variability in accumulation of NH_3_ is determined by a combination of the hindgut fermentation of carbohydrates, as well as an ideally indigestible CP supply. In this respect, Bas and CL diets were not associated with lower hindgut ammonia accumulation than the control, despite their enhanced hindgut VFA production, as these diets reduced ileal CP digestibility, thereby increasing CP supply to the hindgut. As such, there might be potential to mitigate ammonia emissions through dietary inclusion of fiber-rich feedstuffs, provided that ileal CP digestibility is not impaired while hindgut fermentation is enhanced. To effectively assess this, an *in vivo* experiment is needed. In the current experiment, nine fiber-rich ingredients have been incorporated in a complete diet, ranging from 5.0% to 11.4% (on a DM basis), which could be a moderate level based on the fiber source's characteristics and is aimed at testing the interaction of the fiber sources and other dietary ingredients. With the same approach, Gandarillas et al. [27] reported no difference in VFA and ammonia concentration between the control diet and the experimental diets using 15% of Rape and Turnip. Most current studies have tested the fiber digestion and fermentation capacity individually [28,29]. So, further studies with higher levels might have a significant effect on the treatments and could give more evidence for selection sources to use in practical diets.

In conclusion, the chemical composition and in vitro results of the fiber-rich feedstuffs tested here differed significantly; thus, the current findings could help assess the potential nutritive value of unconventional feedstuffs, beyond their chemical composition. When incorporating such fiber sources in complete diets, the enzymatic hydrolysis of CP in the small intestine was lower than for the control diet, indicating that the additivity of nutritive values is not always respected when including such fibrous sources.

## Conclusion

Fiber-rich feedstuffs vary in fiber fraction and chemical composition and differ widely in ileal digestibility and hindgut fermentation. Ileal nitrogen digestibility of diets containing fiber is lower than the control diet.

## List of abbreviations

Bas, banana stem (*Musa acuminata*); Brew, brewery by-product; CL, cassava (*Manihot esculenta*) leaf; CR, cassava root by-product; Caw, cabbage waste (*Brassica oleracea*); Swe, sweet potato vines (*Ipomoea batatas*); Taro, taro leaves and petioles (*Colocasia esculenta*); TF, tofu by-product; Tri, *Trichanthera gigantea* foliage; gm, gram; kg, kilogram; gm/kg, gram per kilogram; mmol/gm, millimole per gram; µmol, micromole; gm CP/kg DM, gram crude protein per kilogram dry matter; gm/kg DM, gram per kilogram dry matter; kJ mol^−1^, kilojoule per mole; h, hour; ml^−1^, per milliliter; gm/l, gram per liter; L, liter; µm, micrometer; FIP-U/gm, fungal inhibitor potency unit per gram; mm, millimeter; M, molar.
